# Chemical Constituents, Antioxidant, Cyclooxygenase Inhibitor, and Cytotoxic Activities of* Teucrium pruinosum* Boiss. Essential Oil

**DOI:** 10.1155/2018/4034689

**Published:** 2018-07-30

**Authors:** Nidal Jaradat, Saad Al-lahham, Murad N. Abualhasan, Abrar Bakri, Haneen Zaide, Jihan Hammad, Fatima Hussein, Linda Issa, Ahmed Mousa, Reem Speih

**Affiliations:** ^1^Department of Pharmacy, Faculty of Medicine and Health Sciences, An-Najah National University, P.O. Box 7, 00970 Nablus, State of Palestine; ^2^Department of Biomedical Sciences, Faculty of Medicine and Health Sciences, An-Najah National University, P.O. Box 7, 00970 Nablus, State of Palestine

## Abstract

**Introduction:**

In traditional medicine, many pharmacological activities have already been ascribed to the genus of* Teucrium* plant. These include antirheumatic antispasmodic, anthelmintic, diuretic, hypoglycemic, and anticancer effects. The recent investigation aimed to characterize and estimate the chemical composition, anti-inflammatory, antioxidant, and anticancer potentials of the essential oil isolated by the microwave-ultrasonic apparatus from* Teucrium pruinosum* leaves collected from Palestine.

**Methods:**

The essential oil (EO) was analyzed by Gas Chromatography equipped with mass spectrometry (GC-MS), while its anticancer activity was evaluated against HeLa cervical adenocarcinoma cells. The ability of* T. pruinosum *EO to inhibit the conversion of Arachidonic Acid (AA) to PGH_2_ by ovine COX-1 and human recombinant COX-2 was determined using a COX inhibitor screening assay. In addition, the antioxidant activity of the EO was evaluated on the basis of the scavenging activity with a stable 2,2-diphenyl-1-picrylhydrazyl (DPPH) method, while Trolox was used as a positive control.

**Results:**

Forty-four molecules were identified in* T. pruinosum *EO, representing 100% of the total EO. Agarospirol was found to be the most abundant component (45.53%) followed by caryophyllene (19.35%). However, the cyclooxygenase inhibitor assay revealed that* T. pruinosum* has potential COX-1 and Cox-2 inhibitory activity with IC_50_ values of 0.25 *µ*g/ml and 0.5 *µ*g/ml, respectively. Moreover, the* T. pruinosum* EO showed moderate antioxidant capacity with an IC_50_ value of 16.98±0.84 *µ*g/ml in comparison with the positive control Trolox, which has an antioxidant potential with an IC_50_ value of 2.09±0.17 *µ*g/ml. In addition, 250, 125, 62.5, 31.25, 15.625, 7.67, and 3.84 mg/ml of* T. pruinosum* EO treatments inhibited mitochondrial activity (cell viability) significantly and extremely by 90-95%.

**Conclusion:**

The current study provided data that revealed that the* T. pruinosum* EO could be a suitable candidate for use as a novel anticancer, anti-inflammatory, and antioxidant medication. Further clinical trials would be required to ensure these effects and to allow the design of suitable pharmaceutical dosage forms from this natural oil.

## 1. Introduction

In ancient times, antecedents utilized plants and other natural products for many purposes such as food, adornment, clothes, soaps, cosmetics, and medicinal uses [1]. Essential oil-bearing plants were found to be useful in traditional medicine for treatment and/or prevention of various diseases. Some of these plants included thyme, peppermint, eucalyptus, sage, clove, cinnamon, and many others [2]. Recently, the total annual production of these oils reached more than 45000 tones and more than 100 valuable essential oils are used commercially for their therapeutic actions encompassing antibacterial, antifungal, antiviral, antioxidant, anti-inflammatory, antinociceptive cytotoxic, and many other effects [3, 4]. In addition, huge amounts of them are utilized as odorants in the perfumery industry and also as flavoring agents in the pharmaceutical and food industries [5].

In addition to this, EOs are used widely in aromatherapy, a significant branch of Complementary and Alternative Medicine (CAM). Here it is used for the treatment of several illnesses and is utilized in several forms including inhalations, oral administrations, mouthwashes, and even trans-dermal applications [6]. However, EOs contain various chemical classes such as terpenoids, alcohols, aldehydes, ketones, oxides, phenolic ethers, and hydrocarbons [7].

Antioxidants are bioactive compounds, which have the ability to prevent or delay oxidation and autoxidation processes by decreasing the formation of harmful free radicals. These radicals can cause great damage to cells, tissues, and even, in some cases, whole organs, which can consequently result in deadly diseases [8].

Many phytochemical classes and other microelements have antioxidant potential such as ascorbic acid, tocopherol, carotenoids, flavonoids, anthocyanins, and polyphenols [9]; however, their mechanism of action is mainly based on the inhibition of DNA oxidation, thus preventing lipid oxidation and therefore the buildup of plaques and LDL oxidation, hence reducing the risk of many types of cancer and various cardiovascular diseases [10]. In pharmaceutical and nutraceutical sciences, the free radical scavenging property has become one of the modern industry standards, especially for assessing the antioxidant strength of herbal medicines, foods, and food preservatives [11].

On the other hand, inflammation is a useful and normal response to fighting infections, leading to a cascade of events; vascular changes and release of chemicals that help in the destruction of harmful agents at the site of injury and repair the damaged tissue. It is recognized as swelling, redness, pain, and heat; however, severe inflammation must be decreased to avoid further destruction in the tissues. At this stage, anti-inflammatory agents have used to block the action of phospholipase or cyclooxygenase (cox1/cox2) by steroids or nonsteroidal anti-inflammatory agents [12]. Nonsteroidal anti-inflammatory medications (NSAIDs) are considered one of the most commonly used classes of drugs worldwide. An estimate of more than 30 million patients per day used this class of medication for the treatment of various inflammatory diseases [13, 14]. However, apart from the potential anti-inflammatory activity of NSAIDs, they are associated with severe adverse side effects such as bleeding, perforation, gastrointestinal ulcers, and obstructions, which has limited the utilization of NSAIDs [15]. In reality, many plants species have shown potential anti-inflammatory activity; cloves containing eugenol, sage containing carnosic acid, and rosemary (rosmarinic acid) provided protection against inflammation by blocking COX 2 [16].

Recently, the amount of research about the herbals benefits in cancer therapy is rising rapidly. This is paramount as cancer is one of the leading causes of death, where the numbers of patients being diagnosed with cancer is also increasing gradually. The mechanisms with which herbs fight against or prevent cancer are varied and its science complex. Some have an immunomodulatory effect, while others can directly act as a cytotoxic agent [17].

Taking into consideration the above facts, an attempt has been made to screen ethnobotanical herbs as anti-inflammatory agents using COX guided activity; antioxidant and cytotoxicity potentials have also been carried out to supplement the results.

*Teucrium pruinosum* Boiss., a member of Lamiaceae family, has been widely utilized in traditional medicine for several therapeutic purposes. It is a perennial herbaceous plant, which reaches about 30-50 cm height. The leaves are tomentose, grey, and oval shaped, with a 1-2-pinnatisect or -ternatisect into narrow-linear rigid lobes with revolute margins. The corolla has a blue color, and elongated lips also coated with a powdery or waxy bloom. The leaves have a strong aromatic odor and bitter taste [18]. The* T. pruinosum* essential oil has been documented in the traditional medicine for the treatment of gastrointestinal disorders, infected wounds, fever, and cold symptoms [19].

The current study aimed to identify the oily matters of* T. pruinosum* chemical composition using Gas Chromatography-Mass Spectroscopy (GC-MS) method of isolated by new microwave-ultrasonic-reactor extractor apparatus technique. In addition to that, the current study aimed to evaluate the antioxidant, cytotoxicity, and cyclooxygenase inhibitory activities of* T. pruinosum* VO.

## 2. Material and Methods

### 2.1. Chemical Reagents

Antioxidant activity was estimated using the following chemicals; DPPH reagent was used which is chemically known as 2,2-diphenyl-1-1-picrylhydrazyl and was purchased from Sigma-Aldrich (Germany), methanol (local filling), and Trolox which is chemically known as ((s)-6-hydroxy-2,5,7,8-tetramethylchroman-2-carboxylic acid) and manufactured by Sigma-Aldrich (USA).

For cytotoxic activity, phosphate buffering saline made by Sigma RPMI-1640 medium from Sigma-Norwich in the United Kingdom, Pen-Strep solution which is composed of penicillin and streptomycin in concentrations of 10000 unit/ml and 10 mg/ml, respectively, and sold from BI/Israel, L-glutamine solution purchased from Sigma∖UK, and MTS reagent purchased from Promega (USA) were used.

### 2.2. Equipment

Water bath (Lab Tech, BPXOP1001040, Namyangju, South Korea), UV-Visible Spectrophotometer (Jenway 7315, Staffordshire, UK), CO_2_ incubator (Esco, 2012-74317, Changi, Singapore), microplate reader (Unilab, 6000, Mandaluyong, USA), inverted microscope (MRC, IX73, Hong Kong, China), vortex (Heidolph Company, 090626691, Schwabach, Germany), and microwave-ultrasonic reactor extractor (Lab-Kits UM2015042801A, Hong Kong, China) were used.

### 2.3. Collection and Preparing of* T. pruinosum*

The wild* T. pruinosum* plant leaves were collected in May 2017 from Ramallah (Beitillu) and Tulkarm (Kafr Zibad) regions of Palestine. Taxonomical identifications were established by the pharmacognosist Dr. Nidal Jaradat at the Pharmacognosy Laboratory at An-Najah National University and the voucher specimen code was Pharm-PCT-2416. The plant leaves were washed well and then dried in the shade at controlled temperature (25±2°C) and humidity (55±5 RH). After the drying process, the leaves were grounded well by using a mechanical grinder into a coarse powder and transferred into airtight containers with proper labeling for future use.

### 2.4. Isolation of* T. pruinosum* EO

The VO of* T. pruinosum* was isolated using the microwave-ultrasonic method as described by Jaradat* et al*., with minor modifications [20]. Within the isolation process, the powder suspension was exposed to ultrasound- and microwaves to improve the extraction process. A 1 L round-bottom flask containing 100 g of the dried leaf powder was placed in microwave-ultrasonic apparatus. In this flask, the powder was mixed in 500 ml distilled water. Then, the flask was connected with Clevenger apparatus which was placed in the same apparatus. During the isolation process, the power of the microwave extractor apparatus was adjusted at 1000 W. The isolation process was conducted for 15 min at 100°C and was repeated three times for the same plant sample. The obtained EO was collected into a clean beaker, chemically dried, and stored at 2-8°C. The obtained* T. pruinosum* EOs yield average percentage was 1.1% from the dried plant sample.

### 2.5. GC/MS Analysis of the EO

The* T. pruinosum* leaves EO was characterized utilizing the GC-MS methods, analyzed by GC/MS using a Perkin Elmer Clarus 500 GC gas chromatograph equipped with Perkin Elmer Clarus 560 mass spectrometer. The separation was achieved by Perkin Elmer Elite-5 fused-silica capillary column (30 m x 0.25 mm, film thickness 0.25 *µ*m). The column temperature was programmed and arranged from 50°C for 5 min to 280°C at 4°C/min. The flow rate of He as a carrier gas was 1 ml/min kept constant for all the chromatographic run. 0.2 *µ*l neat oil was injected in split mode with a split ratio of 1:50 and at a temperature of 250°C. The sample components were identified by matching their mass spectra with those of the library or those of pure standard components and confirmed by their GC retention times.

### 2.6. Cell Proliferation Assay

HeLa cells were cultured in RPMI-1640 media supplemented with 10% fetal bovine serum, 1% penicillin/streptomycin antibiotics, and 1% l-glutamine. Cells were grown in a humidified atmosphere with 5% CO_2_ at 37°C. Cells were seeded at 2.6 x 10^4^ cells/well in a 96-well plate. After 48 h, cells were confluent and media were changed and cells were incubated with 250, 125, 62.5, 31.25, 15.625, 7.67, 3.84, 1.92, 0.96, 0.767, 0.0767, 0.00767, and 0.000767 mg/ml of* T. Pruinosum* EO for 24 h. Cell viability was assessed by CellTiter 96® Aqueous One Solution Cell Proliferation (MTS) Assay according to the manufacturer's instructions (Promega Corporation, Madison, WI). Briefly, at the end of the treatment, 20 *μ*l of MTS solution per 100 *μ*l of media was added to each well and incubated at 37°C for 2 h. Absorbance was measured at 490 nm.

### 2.7. Determination of COX Inhibition

The ability of the* T. pruinosum *EO to inhibit the conversion of Arachidonic Acid (AA) to PGH_2_ by ovine COX-1 and human recombinant COX-2 was determined using a COX inhibitor screening assay with a kit No. 560131 Cayman Chemical, USA. Cyclooxygenase catalyzes the first step in the biosynthesis of AA to PGH2. PGF2*α* produced from PGH2 by reduction with stannous chloride was measured by enzyme immunoassay. This assay is based on the competition between PGs and a PG-acetylcholinesterase conjugate (a PG tracer) for a limited amount of PG antiserum. The amount of PG tracer that is able to bind to the PG antiserum is inversely proportional to the concentration of PGs in the wells since the concentration of the PG tracer is held constant, while PG concentration varies. This antibody-PG complex binds to an anti-IgG antibody previously attached to the well. The plate is washed with a buffer solution and Ellman's reagent, which contains the substrate of acetylcholinesterase, is added to the well. The yellow product of this enzymatic reaction is determined spectrophotometrically in a Microplate reader at 415 nm. The inhibitory assays were performed in the presence of extracts at different concentrations (0.25 and 0.5 *µ*g compound/ml) or of a commercial anti-inflammatory drug such as celecoxib. The anti-inflammatory effect of the test compounds was evaluated by calculating percentage inhibition of PGE2 production. The test compound concentration causing 50% inhibition of PGE2 release (IC_50_) was calculated from the concentration-inhibition response curve by regression analysis [21].

### 2.8. Antioxidant Procedure

Stock solutions of* T. pruinosum *EO and Trolox were dissolved in methanol at 1 mg/ml concentrations. Each one of these stock solutions was diluted in methanol in the following concentrations: 1, 2, 3, 5, 7, 10, 20, 30, 40, 50, 80, and 100 *μ*g/ml. A fresh solution for DPPH solution was dissolved in methanol at 0.002% w/v concentration. The working solutions of* T. pruinosum *EO were mixed methanol and DPPH at a fixed ratio of 1 : 1 : 1. Meanwhile, a negative control solution was prepared by mixing the above-mentioned DPPH solution with methanol only in 1 : 1 ratio. All above-mentioned solutions were incubated for 30 min at 25°C in a dark place. The UV-visible spectrophotometer at a wavelength of 517 nm was utilized to measure the optical density of all studied samples. The antioxidant activities of* T. pruinosum *EO and for reference compound (Trolox) were estimated by using the following formula:

(1) The  inhibition % of  DPPH  activity=AB−AEAB×100%,

where AB is absorbance of the blank and AE is absorbance of the EO.

The IC_50_ (antioxidant half-maximal inhibitory concentration) of* T. pruinosum* EO and Trolox as well as their standard deviations were calculated by using BioDataFit Edition 1.02 [22].

### 2.9. Statistical Analyses

Determination of the chemical constituents of* T. pruinosum* EO, antioxidant activity, and COX inhibitory was carried out in triplicate for each sample. The obtained results were presented as means ± standard deviation (SD).

## 3. Results

### 3.1. *T. pruinosum* EO Composition

The EO content in the leaves of the native species* T. pruinosum,* growing wildly in Palestine, was found to be 1.1% based on the plant dried weight. The EO was analyzed and characterized using the GC-MS technique. Their identification process was made by comparing their mass spectra retention indices with those given in the literature and authentic samples. Forty-four constituents, representing 100% of the oil, were identified. The major components of the oil were agarospirol (45.53%) and caryophyllene (19.35%) as shown in Table 1.

**Figure 1:**
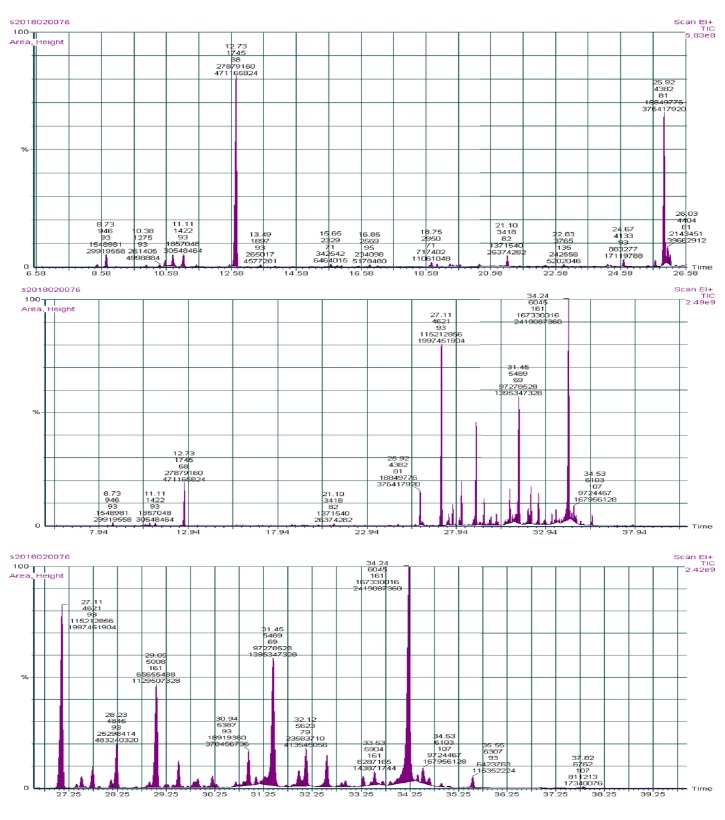
**GC-MS chromatogram of** *T. pruinosum *leaves EO.

The oil of* T. pruinosum *leaves comprised sesquiterpenoid (93.27%), monoterpenoid (4.04%), and other phytochemical compounds (2.69%).  Figure 1 shows the gas chromatogram of T.* pruinosum *leaves EO.

### 3.2. COX Inhibitory Activity

The enzyme inhibition activity of* T. pruinosum* EO was carried out using an ELISA kit. The calculated percentage inhibition for the tested compounds is shown in Table 2. The results showed that the* T. pruinosum* EO demonstrated a selective inhibition activity toward COX-2 enzyme. The calculated IC_50_ was found to be 0.103 *µ*g/ml. However,* T. pruinosum* EO also showed inhibition activity toward COX-1 enzyme and the calculated IC_50_ was found to be 0.208 *µ*g/ml.

### 3.3. Cytotoxic Effect

**Figure 2:**
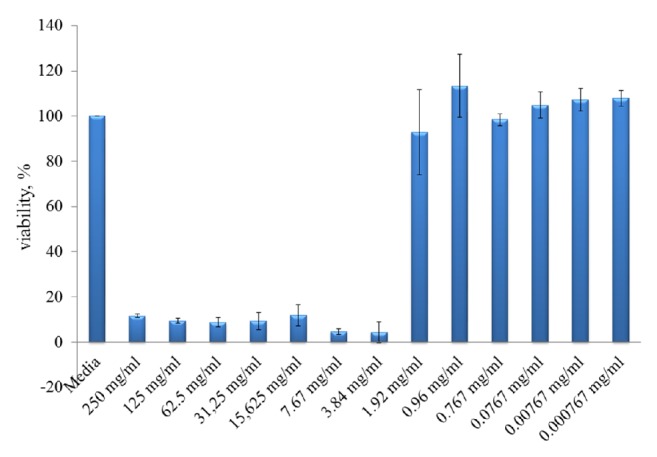
MTS assay of* T. pruinosum* EO.

MTS assay was used to determine the cytotoxicity effect of* T. pruinosum* on HeLa cervical adenocarcinoma cells. As shown in (Figure 1), 250, 125, 62.5, 31.25, 15.625, and 7.67 mg/ml of* T. pruinosum* EO treatment inhibited mitochondrial activity (cell viability) significantly and extremely by 90-95% as presented in Figure 2.

### 3.4. Antioxidant Activity

**Figure 3:**
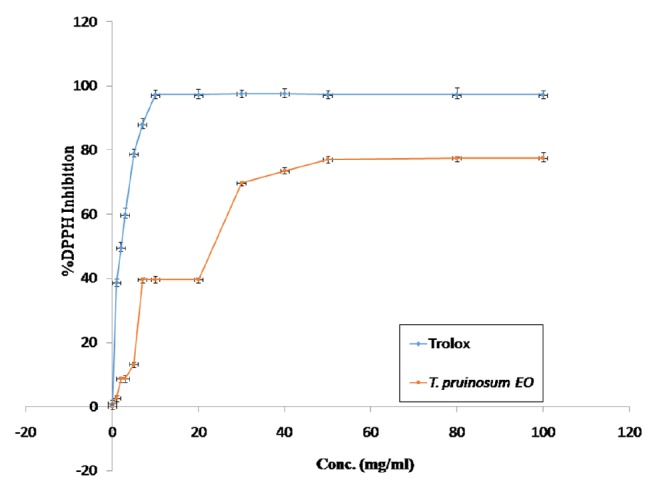
DPPH inhibitory activity of* T. pruinosum *EO in comparison with Trolox.

To calculate IC_50_ logarithmic curve was made for the original curve then added the trend line from which was obtained the required equation from which IC_50_ values were calculated as revealed in Table 3 and Figure 3. However, the IC_50_ for Trolox was calculated using the following equation; y = 37.96x + 37.73, from which IC_50_ value was 2.09±0.17 *µ*g/ml.

While the IC_50_ value for* T. pruinosum* EO was calculated using the following equation: y= 43.58x - 4.030, from which IC_50_ was 16.98±0.84 *µ*g/ml.

## 4. Discussion

The utilization of traditional herbal medicine has received huge interest worldwide. This renewed interest in medicinal plant research has focused on herbal products, which have traditional therapeutic potentials. However, the use of natural products in the discovery of medicines has remained an important issue in the investigation of novel pharmaceutical preparations [23].

In traditional medicine, many pharmacological activities have already been ascribed to the genus of* Teucrium* plant and among them antirheumatic, antispasmodic, anthelmintic, diuretic, antidiabetic, and anticancer effects [19, 24, 25].

Initially, the EO of* T. pruinosum *was isolated using a newly established technique (microwave-ultrasonic-extractor reactor) and the EO yield was 1.1% of the dried herb weight. Forty-four compounds were identified in the EO of* T. pruinosum* and represented 100 %, of which the major compounds were agarospirol (45.53%) and caryophyllene (19.35%); other compounds were less than 4%.

Agarospirol (Hinesol) is a unique alcohol sesquiterpenoid EO isolated from the Chinese* Atractylodes lancea* and* Aquilaria agallocha *medicinal plants [26].

Many previously conducted studies aimed to identify the chemical compositions of* Teucrium* genus EOs using the GC-MS method. For example, the major EO components of* Teucrium flavum* growing spontaneously in Tunisia which was extracted by a hydrodistillation method were *β*-caryophyllene (32.5%) and *α*-humulene (17.8%) [27].

Another study identified the chemical composition of the EO obtained from Teucrium marum and the main components were isocaryophyllene (20.24%), *β*-bisabolene (14.73%), *β*-sesquiphellandrene (11.27%), *α*-santalene (10.97%), dolichodial (9.38%), and *α*-caryophyllene (7.18%) [28].

A study conducted by Cozzani* et al*. characterized the chemical components of* Teucrium polium* subsp.* capitatum* (L.) EO from Corsica and the major components were *α*- pinene (28.8%), *β*-pinene (7.2%), and p-cymene (7.0%) [29].

However, another investigation performed by Menichini*et al*. identified the chemical constituents of the* Teucrium brevifolium, Teucrium flavum, Teucrium montbretii,* and* Teucrium polium *EOs and the most abundant components were spathulenol and D-cadinene; caryophyllene and 4-vinyl guaiacol; carvacrol and caryophyllene oxide; and carvacrol and caryophyllene, respectively [30].

The current study results clearly demonstrated that the EO of* T. pruinosum *plant has more selective inhibition towards COX-2 enzyme. The respective mean IC_50_ values for COX-1 and COX-2 and the COX-2/COX-1 ratio of the current result were 2.02.

However, Kato* et al*. showed that COX-2/COX-1 ratio of the inhibition of the COX-2 selective NSAID of etodolac has a ratio of 1.9, while nonselective NSAID like ibuprofen has a ratio of 0.15 [31].

These results demonstrated that the EO of* T. pruinosum* has potential anti-inflammatory activity almost the same as the NSAID drug etodolac.

Moreover, the antioxidant activity of the EO of* T. pruinosum *was estimated by using the DPPH method which is one of the most commonly used assays due to its simplicity and high sensitively [32]. The established results of the current study revealed that the DPPH method antioxidant results revealed that the EO of* T. pruinosum *has antioxidant potential with an IC_50_ value of 16.98±0.84 *µ*g/ml in comparison with the positive control molecule (Trolox) which has an IC_50_ value of 2.09±0.17 *µ*g/ml.

In an investigation performed by Hammami* et al*., on the antioxidant activity of the EO of another species of* Teucrium* plant (*T. pseudochamaepitys*) using DPPH method showed that this EO had antioxidant activity with an IC_50_ value of 51.89±1.00 *µ*g/ml [33].

Other researchers found that the antioxidant activity of* Teucrium polium*,* Teucrium marum*,* Teucrium sauvagei,* and* Teucrium orientale *EOs using DPPH method and the IC_50_ values were 90±1.89 *µ*g/ml, 65.65±2.78 *µ*g/ml, 100±0.59 *µ*g/ml, and 250±1.5 *µ*g/ml, respectively [28, 34–36].

Several types of the EOs possessed antioxidant activity in addition to the ability of them possessing anti-inflammatory effects; peppermint and chamomile EO_S_ have anti-inflammatory and antioxidant agents [37].

Many of the available pharmaceutical formulations, which are indicated for the treatment of rheumatism and other inflammatory conditions, contain EO, isolated from eucalyptus, camphor, peppermint clove, and many others [38].

In addition, antioxidant molecules play an essential role in inflammatory reactions. The free radicals and the reactive oxygen species can cause oxidative stress in the cells leading to inflammatory and infectious conditions. In addition, oxidative stress can cause harmful damage to the cells and tissues, which in turn augments the state of inflammation [39].

The current study showed that the EO of* T. pruinosum *possessed moderate antioxidant capacity with an IC_50_ value of 16.98±0.84 *µ*g/ml in comparison with the positive control Trolox, which has antioxidant potential with an IC_50_ value of 2.09±0.17 *µ*g/ml.

A study established by Masuda* et al*., in which agarospirol molecule was identified and isolated from the EO of* Atractylodes lancea*, showed that this molecule inhibited cell growth and induces apoptosis in human leukemia HL-60 cells [40].

Moreover, Stankovic* et al.* showed that among the investigated extracts of* T. chamaedrys*,* T. montanum*,* T. arduini*, and* T. scordium* subsp.* scordium* all extracts significantly reduced cell viability in a dose-dependent manner with very low IC_50_ values. The highest content of phenolic compounds and the best cytotoxic activity on HCT-116 cells after 24 h of exposure was in* T. chamaedrys *extract, with IC_50_ values of 5.48 *μ*g/ml after 72 h, while the methanolic extract of* T. arduini* appeared to have the best cytotoxic activity on HCT-116 with IC_50_ values of 0.37 *μ*g/ml [41].

Another investigation established by Ali* et al.* showed that crude saponins of* Achillea wilhelmsii* and* T. stocksianum* had cytotoxic activity with LC_50_ values 2.3±0.16 and 5.23±0. 34 *μ*g/ml, respectively [42].

In addition, a study conducted by Menichini* et al*. showed that the most antiproliferative oils were those from* T. polium* ssp.* capitatum* and* T. montbretii* ssp.* heliotropiifolium* on CACO-2 cell lines (IC_50_ = 52.7 and 92.2 *μ*g/ml, respectively). The* T. brevifolium* EO showed a selective cytotoxicity on COR-L23, while significant activity was exerted by* T. polium* oil on C32 [30].

Briefly, the current study identified for the first time the chemical composition of* T. pruinosum *EO from Palestine and the pharmacological screenings showed that the EO of* T. pruinosum *possessed moderate antioxidant capacity with IC_50_ value of 16.98±0.84 *µ*g/ml in comparison with the positive control Trolox which has antioxidant potential which had IC_50_ value of 2.09±0.17 *µ*g/ml. In addition, the EO of* T. pruinosum* had potential anti-inflammatory activity almost the same as the NSAID drug etodolac, while a 7.67 mg/ml of* T. pruinosum* EO treatment inhibited mitochondrial activity (cell viability) significantly and extremely by 90-95%.

A study conducted by Masuda* et al*. found that agarospirol had an anticancer effect and this effect mainly associated with apoptosis potentials such as nuclear fragmentation and DNA fragmentation. Furthermore, it was reported in this study that agarospirol induced activation of the c-Jun N-terminal kinase (JNK), but not p38, prior to the onset of apoptosis. These results suggested that agarospirol induced apoptosis through the JNK signaling pathway. Therefore, agarospirol may represent a novel medicinal drug having indications in the treatment of various types of cancers [40].

Further in vivo clinical trials required to approve or disapprove these results and to determine the suitable pharmaceutical dosage form and therapeutic dose.

## 5. Conclusion

The current investigation is the first to evaluate the chemical constituents of* T. pruinosum *EO, also the first one which investigated the anticancer, anti-inflammatory, and antioxidant activities of the studied EO employing a variety of in vitro assays. The reported data in the current study showed that the EO of* T. pruinosum *possessed the highest content in alcoholic sesquiterpenoid namely agarospirol. The remarkably high content of agarospirol could be of interest for further phytochemical and biological investigation of* T. pruinosum*, taking into account the fact that agarospirol-rich oils showed marked anticancer activity. In summary, our results indicated that* T. pruinosum* EO induced apoptosis effect against HeLa cervical adenocarcinoma cells line and, in addition, has cyclooxygenase inhibitory and antioxidant activities suggesting the possibility that* T. pruinosum* EO may be a useful anticancer, antioxidant, and anti-inflammatory drug that could enhance the therapeutic efficacy of commercially available drugs. Further studies are needed to clarify the details of the mechanisms of action for* T. pruinosum* EO which induced apoptosis.

## Figures and Tables

**Table 1:** The chemical composition of* T. pruinosum *EO.

Name	Rt	RI	Area	%, ±SD
Agarospirol	33.525	900	342947197	45.53±0.33
Caryophyllene	27.107	923	145768068	19.35±0.23
D-Limonene	12.727	916	27879160	3.70±0.02
Alpha Caryophyllene	28.233	905	25298414	3.36±0.03
Caryophyllene oxide	32.124	786	23583710	3.13±0.01
Seychellene	27.413	847	22445657	2.98±0.02
Di-EPI-Alpha-cedrene	30.954	827	21010945	2.79±0.01
Guaiol	32.55	896	20393386	2.71±0.03
Isoledene	29.048	829	19870923	2.64±0.01
Beta humulene	34.525	882	19448934	2.58±0.02
Alpha-Bourbonene	25.912	855	18849776	2.50±0.01
(-)-Spathulenol	31.974	790	12395272	1.65±0.001
Alpha-Cubebene	30.204	870	6297936	0.84±0.002
Trans-Alpha-Bergamotene	27.518	846	5787941	0.77±0.003
Thujopsene	35.655	785	4732613	0.63±0.001
Cyclohexane, butylidene	32.93	769	3402058	0.45±0.001
(-)-Alpha-Panasinsen	34.415	855	3116025	0.41±0.001
Humulene-(V1)	29.268	903	3034941	0.40±0.001
Alpha-Santalol	30.844	797	2755883	0.37±0.001
(-)-Isolongifolol, methyl ether	32.85	777	2420032	0.32±0.001
Arachiddonic acid	26.027	780	2143451	0.28±0.001
Longifolene-(V4)	33.7	879	2091585	0.28±0.001
Beta-Vatirenene	34.906	836	2075653	0.28±0.001
Beta-Pinene	11.101	788	1857048	0.25±0.001
1-Octen-3-Ol	10.781	828	1676174	0.22±0.001
Santalol	31.869	824	1608692	0.21±0.001
1R-Alpha Pinene	8.725	910	1548981	0.21±0.001
(1R)-(-)-Myrtenal	21.095	780	1371540	0.18±0.001
Ocimene	19.314	841	1174574	0.16±0.001
Vitamin A aldehyde	31.719	848	1130310	0.15±0.001
3-Carene	10.546	865	1058024	0.14±0.001
Androstan-17-one,-ethyl-3-hydroxy-(5alpha)	32.76	832	794975	0.11±0.001
Ylangene	25.642	859	716756	0.10±0.001
2-Cyclohexen-1-Ol, 2methyl-5-(1-methyl ethynyl)- cis	20.225	855	515827	0.07±0.001
Alpha Phellandrene	8.45	890	349106	0.05±0.001
3-Octanol	11.491	761	338021	0.04±0.001
Beta Phellandrene	10.376	859	261405	0.03±0.001
1,3,8-P-Menthatriene	12.527	875	241672	0.03±0.001
Ethyl M-Tolyl Carbinol	19.63	754	238282	0.03±0.001
Benzaldehyde	9.946	916	236240	0.03±0.001
Methyl 7,11,14 eicosatrienoate	17.574	758	105052	0.01±0.001
Cyclohexanol, 2-Methylene-5-(1-methylethenyl)	19.069	752	79564	0.01±0.001
Gamolenic acid	16.513	677	67519	0.01±0.001
(+)-4-Carene	15.038	814	42967	0.01±0.001

Total			753162289	100.00

Phytochemical classification				
Sesquiterpenoid hydrocarbon				39.35
Sesquiterpenoid oxide				3.13
Sesquiterpenoid alcohol				50.79

Total sesquiterpenoid				93.27

Monoterpenoid hydrocarbon				4.04
Other phytochemical molecules				2.69

Total phytochemical classes				100.00

**Table 2:** COX enzyme inhibition activity.

Sample concentration	% of COX_2_ inhibition, ±SD	% of COX_1_ inhibition, ±SD
0.25	95.12713±1.01	80.1±0.97
0.5	97.01654±1.13	99±1.21

**Table 3:** Free radical scavenging activity and IC_50_ values of *T. pruinosum *EO and Trolox.

Conc., (*µ*g/ml)	% inhibition of DPPH by Trolox	±SD	% inhibition of DPPH by *T. pruinosum *EO	±SD
0	0	0	0	0
1	38.6	1.23	2.64	0.82
2	49.5	1.75	8.68	0.42
3	59.8	2.23	8.68	1.03
5	78.8	1.62	13.2	0.62
7	88	2.1	39.62	0.62
10	97.2	1.52	39.62	1.03
20	97.2	1.85	39.62	0.62
30	97.5	1.3	69.81	0.62
40	97.5	1.82	73.58	1.03
50	97.2	1.34	77.11	1.03
80	97.2	2.25	77.52	0.41
100	97.2	1.4	77.52	1.85

IC_50_ value, (*µ*g/ml)	2.09	0.17	16.98	0.84

## Data Availability

The data used to support the findings of this study are included within the article.
